# The influence of intersensory discrepancy on visuo-haptic integration is similar in 6-year-old children and adults

**DOI:** 10.3389/fpsyg.2014.00057

**Published:** 2014-01-30

**Authors:** Bianca Jovanovic, Knut Drewing

**Affiliations:** Department for Developmental Psychology, Institute for Psychology, Justus-Liebig UniversityGiessen, Germany

**Keywords:** multisensory, child development, integration, visuo-haptic display, intersensory integration

## Abstract

When participants are given the opportunity to simultaneously feel an object and see it through a magnifying or reducing lens, adults estimate object size to be in-between visual and haptic size. Studies with young children, however, seem to demonstrate that their estimates are dominated by a single sense. In the present study, we examined whether this age difference observed in previous studies, can be accounted for by the large discrepancy between felt and seen size in the stimuli used in those studies. In addition, we studied the processes involved in combining the visual and haptic inputs. Adults and 6-year-old children judged objects that were presented to vision, haptics or simultaneously to both senses. The seen object length was reduced or magnified by different lenses. In the condition inducing large intersensory discrepancies, children's judgments in visuo-haptic conditions were almost dominated by vision, whereas adults weighted vision just by ~40%. Neither the adults' nor the children's discrimination thresholds were predicted by models of visuo-haptic integration. With smaller discrepancies, the children's visual weight approximated that of the adults and both the children's and adults' discrimination thresholds were well predicted by an integration model, which assumes that both visual and haptic inputs contribute to each single judgment. We conclude that children integrate seemingly corresponding multisensory information in similar ways as adults do, but focus on a single sense, when information from different senses is strongly discrepant.

## Introduction

Perception is essentially multimodal, with different senses contributing different aspects to the overall appearance of the environment. In some cases, different senses can also convey redundant information about the same object property, as for example, size: the size of an object can be seen and felt at the same time. While it is obvious that being able to process different information enriches our perception, it is at first glance less clear how the convergence of redundant information from different senses contributes to perception. Well-established models on perceptual integration [overview in Ernst and Bülthoff (2004)] suggest that adults combine redundant information from different senses in a way that enhances the reliability of the resulting percept. In contrast, studies with 5- to 6-year-old children imply that their judgments are dominated by one sense, that is, for example, either by visual or by haptical information, depending on testing conditions (Misceo et al., 1999; Gori et al., 2008). A corresponding dominance has been suggested to reflect a lack of multisensory integration (Gori et al., 2008). This conclusion, however, stands in contrast to studies with infants implying that the integration of spatially and temporally coordinated information from different senses begins during the first year of life already (e.g., Rosenblum et al., 1997; Kerzerho et al., 2008). The present paper investigates one possible alternative explanation for the failure to find integration in the 5- to 6-year-old children. We argue that in the studies finding unisensory dominance in children (Misceo et al., 1999; Gori et al., 2008) the ways in which stimuli were presented might have suggested to the children that the information provided by the different senses did not originate from one and the same object, and thus children did not relate the inputs.

How do adults combine redundant information from different senses? A model that has been widely applied to different instances of information integration in human perception is the now well-established Maximum-Likelihood-Estimate (MLE) model of “optimal integration” (Landy et al., 1995; Ernst and Bülthoff, 2004). According to this model the brain takes into account all perceptual information (or cues) available to judge a property, e.g., size information from different senses for size judgments, and combines them in order to obtain a maximally reliable percept. In a first step, estimates ($\hat{s}$_*i*_) for the property are derived from each cue (*i*) and in a second step, by weighted averaging, all estimates are combined into a coherent percept ($\hat{s}$_*P*_):

(1) $${\hat{s}}_{P}=\sum_{i}w_{i}{\hat{s}}_{i}\text{ with }\sum_{i}w_{i}=1;0\leq w_{i}\leq 1$$

Estimates derived from each perceptual cue are prone to noise (variance σ^2^_*i*_). By averaging different estimates, the system can reduce the variance in the combined percept (Landy et al., 1995). How the weights are set, depends on the reliability of the individual estimates. The reliability is the inverse of the variance (=reliability *R*_*j*_ = 1/ σ^2^_*j*_). “Optimal” cue weights *w*_j_ that result in the minimal variance σ^2^_*P*_ of the final percept $\hat{s}$_*P*_ are proportional to the relative reliabilities of the estimates (Oruç et al., 2003):

(2) Wj=Rj∑i=1..,j,…N​​​​​​​​Ri  with Rp=∑i=1..NRi

Accordingly, dominance of a single sense results when the variance of the estimate derived from that sense is rather low as compared to the variance of the other senses' estimates. If the variances are similar, the different cues are predicted to have similar contributions to each perceptual judgment. Predictions from the MLE model have been confirmed for the integration of different cues within a single sense, such as different visual depth cues like shading or stereo cues (e.g., Young et al., 1993; Perotti et al., 1998; Backus et al., 1999; Hillis et al., 2004). Concerning multisensory integration, the predictions of the MLE model have been tested in several studies (e.g., Ernst and Banks, 2002; Alais and Burr, 2004; Helbig and Ernst, 2007a). In these studies variances of the single senses' estimates on a property, such as object size, have been assessed by measuring discrimination thresholds for that property in a condition in which subjects were using a single sense alone. The single senses' actual variances were, then, used to predict the expected optimal variances and weights of the estimates in a bisensory condition. Actual bisensory variances were assessed by measuring discrimination thresholds in bisensory conditions, and the senses' weights were assessed by introducing small unnoticeable intersensory discrepancies between the information given in the two senses on the same object, e.g., discrepancies between seen and felt length of that object. Many studies quantitatively confirm that multisensory integration is well described by the assumptions of MLE model, including the optimal weighting of information (Ernst and Banks, 2002; Alais and Burr, 2004; Helbig and Ernst, 2007a). However, there is controversy over the situations in which the weights are indeed set “optimally” (Oruç et al., 2003; Rosas et al., 2005; Cellini et al., 2013). Thus, suboptimal integration has also been observed, for example, for the integration of signals to slant from visual texture cues and cues from haptic exploration (Rosas et al., 2005). In a few studies predictions from integration models have been contrasted with predictions from the model of “probabilistic cue switching” (or “stochastic selection”; e.g., Nardini et al., 2008; Serwe et al., 2009; Kuschel et al., 2010). Probabilistic cue switching means that participants do not integrate the cues in multi-cue situations, but focus on one single cue of a given stimulus per trial, with a constant relative choice probability for each cue (hence, “probabilistic”). That is, which one of several cues is used for a perceptual judgment, alternates between stimulus presentations. This contrasts with integration, where each of the available cues contributes to the judgment on each stimulus presentation. The model of probabilistic cue switching has proven useful to identify conditions under which cues are not integrated, which, for instance, has been observed when cues are related to each other only on a symbolic level (Serwe et al., 2009).

Developmental studies have examined whether children combine multisensory information in similar ways as proposed for adults in the integration models. Psychophysical measurement is applicable only with children who are able to give verbal judgments on the object properties in question and to compare these in a systematic way. Thus, the relevant studies with children have mainly focused on children from the age of 5 years and older. The findings from the relevant child studies are mixed. Supporting evidence that is in line with the adult findings comes from a study by King et al. (2010). They measured 7- to 13-year-old children's integration of visual and proprioceptive cues on target position. Children's estimates of target position were influenced by both, visual and proprioceptive cues, and the weight of proprioception increased with age. This increase was linked to age-related improvements in proprioceptive precision. The pattern of findings was interpreted as being consistent with the assumption that information is weighted more strongly the more reliable it is. However, the authors did not test directly for integration mechanisms, as they had not enough data. In contrast, Misceo et al. (1999) found little evidence for integration in a visuo-haptic matching task. These authors used an anamorphic lens to induce an intersensory discrepancy between the observed and the felt size of an object. Children aged 6, 9, and 12 years viewed objects through a lens while manually grasping them through a hand-concealing cloth. Then, they selected a match from a set of comparison objects. When adults performed this task (Hershberger and Misceo, 1996), the size of the match was in-between the observed and the felt object size. For adults, the size of the match deviated from the felt size by 30–70% of the discrepancy between the seen and the felt size, which corresponds to a visual weight of 30–70% in the judgment. Six-year-old children, however, exhibited nearly complete visual dominance (about 80% visual weight; Misceo et al., 1999). In another recent developmental study on visuo-haptic integration (Gori et al., 2008) 5- to 6-year-old children (but not adults) were again found to display almost complete unisensory dominance: haptic dominance in a size discrimination task (~20% visual weight, age group 5 years) and visual dominance in an orientation discrimination task (~90% visual weight, age group 6 years). In addition, Gori et al. (2008) tested the predictions of the MLE model of optimal integration. For the 5- to 6-year-old children both weights and bisensory variances clearly deviated from the model's predictions. In the age groups between of 8 and 10 years, however, the children's behavior increasingly resembled that of adults, suggesting that the ability to integrate visual and haptic input develops during this period. In contrast, the data from the 5- to 6-year-olds were interpreted as indicating that children of this age do not yet integrate information from different senses, but rather rely on one single sense (Gori et al., 2008).

This is quite surprising, given the evidence that even infants are able to relate information originating from different senses [overview in Lickliter and Bahrick (2004)]. One example of early multisensory integration is the McGurk effect (McGurk and MacDonald, 1976), which shows that the combination of multisensory information can lead to a percept that is qualitatively different from that provided by the single senses: when participants are presented with discrepant auditory and visual syllables, often some kind of fusion occurs between the syllables (Rosenblum et al., 1997). Rosenblum et al. (1997) have found that the McGurk effect is already present in 5-month-old infants (see also Kushnerenko et al., 2008). The McGurk effect has also been found in 4- to 10-year-old children with similar effects as in adults, or, in some cases, somewhat smaller than in adults (e.g., Massaro et al., 1986). In the same vein, recent work indicates that visual speech cues can help infants to discriminate phonemes (Teinonen et al., 2008). Finally, and of crucial importance, Kerzerho et al. (2008) showed that 5-month-old infants' discrimination of different haptically experienced orientations can be influenced by the presentation of consistent or discrepant visual context cues: when the visual context cues were consistent with the haptic cues, infants became able to differentiate between orientations they were unable to differentiate with haptic information alone. In contrast, when the spatial orientation presented visually was discrepant to the one presented haptically, infants' performance was disrupted.

Given that there is evidence of infants' ability to match and integrate perceptual information from different senses, it is puzzling that multisensory integration is so hard to find in older children. On the one hand, it could be the case that these early abilities rely on qualitatively different mechanisms for processing and integrating perceptual information. On the other hand, children's low integration performance in the studies cited above (Misceo et al., 1999; Gori et al., 2008) could be explained by several methodological issues. As an example, Misceo et al. (1999) used a lens to introduce intersensory discrepancy. However, the lens used in these studies was relatively strong, so that it halved the visually presented object size. Stimuli with correspondingly large discrepancies might not induce natural multisensory processes, because large discrepancies provide a cue suggesting that the information from the different senses probably stems from different objects and does not belong together. In the study by Gori et al. (2008), this specific problem was avoided, as the induced discrepancies were quite small. However, the objects used in that study consisted of two spatially divided parts. Thus, participants examined a pair of objects attached to the front and rear surfaces of a panel so as to simulate a single object protruding through a hole. The participants felt the one in the back while viewing the one in front. Crucially, with this method it might not have been apparent to the participants that haptic and visual inputs stemmed from the same object, as they did not originate from the same location. Earlier work on the bi-partite task has shown that even for adults task instructions regarding a shared origin of visual and haptic inputs is required to promote integration (Miller, 1972). Correspondingly, Gepshtein et al. (2005) demonstrated that physical proximity is an important precondition for the combination of different sensory cues.

Taken together, in the studies discussed here (Misceo et al., 1999; Gori et al., 2008), the cues provided by the experimental paradigms might have hindered the young children to perceive a relation between the visual and the haptic inputs, as on the one hand there were great size discrepancies between seen and felt stimuli (Misceo et al., 1999) and on the other hand the physical proximity between felt and seen parts of the stimuli was low (Gori et al., 2008). In the present studies, we sought to overcome these various methodological problems.

We studied visuo-haptic length judgments using the lens technique, because this technique allowed us to provide the participants with perceptual cues that indicate the common origin of haptic and visual inputs: while participants looked at the stimulus, they simultaneously saw how their fingers touched the stimulus through a soft cloth, which should be a strong cue indicating that they felt and saw the same single object (Helbig and Ernst, 2007b). Participants touched through a soft cloth in order to insure that they were able to see their finger movements without any optical distortion of their fingers. To test for the influence of magnification, we used different lenses. The lenses induced either large or small visuo-haptic discrepancies. We expected that with small intersensory discrepancies even 6-year-old children would be able to use inputs from both senses, much in the same way as adults do. Beside adults, we decided to investigate 6-year-old children, because the previous studies (Misceo et al., 1999; Gori et al., 2008) suggest that children in this age group can understand and accomplish the necessary experimental tasks, while at the same time their abilities to integrate visual and haptic information are not yet developed. If we however found positive evidence of integration already in 6-year-old children, the assumption that integration abilities do not develop before school age would be cast into doubts. We tested behavior against predictions from models of optimal and suboptimal integration and of probabilistic switching between the senses.

## Materials and methods

Six-year-old children and adults compared the length of different rectangular standard stimuli (of 20–30 mm length) with a set of comparison stimuli in a two-interval forced choice task combined with the method of constant stimuli. Standard stimuli were presented either haptically (precision grip), or visually, or to both senses. Comparison stimuli were presented only haptically in each condition. In visuo-haptic conditions we used cylindrical reducing and magnifying lenses in order to dissociate the seen length of the standard stimulus from its felt length. For the groups with large discrepancies between seen and felt length the magnifying/reducing factor was 1.5; for the small-discrepancy groups the factor was 1.25. Due to their cylindric shape the lenses did not affect the seen width of the objects.

Participants successively explored the standard and the comparison stimulus and afterwards had to indicate which of the two stimuli they had perceived to be larger. We assessed length judgments of the standard stimuli by the points of subjectively equal length (PSE). From visuo-haptic length judgments we derived the senses' weights in bisensory judgments. In addition, we measured 84%-discrimination thresholds (just noticeable difference, JND) in order to assess uni- and bisensory variances. We predicted bisensory weights and thresholds using models that assume optimal integration, suboptimal integration or no integration at all (probabilistic switching between senses).

In contrast to most of the previous studies (e.g., Ernst and Banks, 2002; Gori et al., 2008), in which comparison stimuli were presented in the same modality as the standard stimuli, in the present experiment the comparison stimuli were always presented only haptically. Our modified method measured values JNDs and PSEs on the same scale in all modality conditions, namely as compared to haptic stimuli, and differential biases between the senses were assessed and considered in the further analyses [cf. Equation (3) and footnote 1; cf. also (Reuschel et al., 2010)]. In contrast, the previously used methods measured these values on different modality-specific scales and did not assess potential biases. Second, it has been argued that automatic aspects of multisensory integration are better captured when participants compare bisensory stimuli to unisensory comparison stimuli, as bisensory comparison stimuli can also trigger deliberate processes of integration (cf. e.g., Shams et al., 2000; Bresciani et al., 2006; Ernst, 2006; Helbig and Ernst, 2007b). Previous adult studies, in which bisensory stimuli were matched to unisensory stimuli, show, however, that judgments can be slightly shifted toward the sense to which the comparison stimuli were presented (Hershberger and Misceo, 1996; Helbig and Ernst, 2007b). Ernst (2006) explains this shift by an incomplete fusion between the senses, while with complete fusion judgments on bisensory stimuli that refer to either of the two senses are predicted to be the same [corresponding findings in Lederman et al. (1986)]. If fusion is complete, findings are consistent with the predictions of the MLE model on optimal integration, while incomplete fusion corresponds to suboptimal automatic integration.

### Participants

Children were sampled from different kindergartens in the regions of Hagen and Giessen, adult participants were mainly sampled from Giessen University. Informed consent was obtained from the parents before testing. We collected complete data sets from 40 adults and 40 children. However, we removed the data from 10 participants (7 children, 3 adults), who had an outlier JND defined by a measured value larger/smaller than average ±3 standard deviations in the respective condition. Given the small number of data points per JND a temporary lack of attention to the task is a potential reason for such outliers. The final sample of the large discrepancy group included 22 children with a mean age of 6;2 [years; months] and an of age range 5;5–6;11 (50% females; 68% right-handed) and 24 adults with a mean age of 32 years and an age of range: 18–51 years (46% females; 79% right-handed). The final sample of the small discrepancy group included 11 children with a mean age of 5;6 and an age range of 5;1–5;11 (36% females; 73% right-handed) and 13 adults with a mean age of 25 years and an age range of 20–34 years (62% females; all right-handed).

### Apparatus and stimuli

The entire apparatus was mobile and the experiments were conducted in a quiet room in the respective kindergartens, or the university. Participants sat–vis-à-vis to the experimenter–in front of a table. Side-by-side, on the top of the table stood two “presentation boxes.” One presentation box contained the standard stimulus, the other one contained the comparison stimulus. Participants could look at the stimulus through diving goggles and an exchangeable lens; a blind in the box occluded left-eye views. The experimenter placed one stimulus at the bottom of each box. After placement, participants could reach through a soft cloth at the sides of the box to simultaneously see and feel the stimulus. The soft cloth prevented participants from seeing their fingers through the lens while they were able to see their finger movements. Stimuli were rectangular plastic plates that were covered with a red-colored smooth film (1 mm high, 20 mm wide, length 14–36 mm). A custom-made computer program prescribed the presentation order and collected the participants' responses.

### Design and procedure

The design comprised the between-participant variables Age Group (Children vs. Adults) and Intersensory discrepancy (large vs. small) and the within-participant variables Modality (Haptics alone, Vision alone, Haptics and Vision) and Stimulus Set (short-magnified vs. long-reduced). Each of the 12 combinations of the conditions of the variables Intersensory discrepancy, Modality, and Stimulus Set was realized by a specific “standard stimulus,” as we will explain below in detail.

Each standard stimulus was paired with a range of comparison stimuli that were presented haptically in each condition several times—using the method of constant stimuli in a two-interval forced choice paradigm. In each trial we presented the participants successively with a standard and a comparison stimulus. Participants were instructed to indicate which stimulus had been larger by pointing to the corresponding presentation box. From these responses we calculated the individual points of subjective equality (PSE) and the 84%-discrimination thresholds (just noticeable difference, JND) of each standard compared to the comparison stimuli.

The 12 different standard stimuli were implemented using two different physical stimuli; a shorter physical stimulus that was visually presented via a magnifying lens (Stimulus Set: short-magnified) and a longer physical stimulus that was visually presented via a reducing lens (Stimulus Set: long-reduced). For the large discrepancy condition the physical stimuli were 20 and 30 mm long so that haptic standard stimuli in the large discrepancy condition were 20 and 30 mm long. For visual presentation the 20 mm-stimulus was magnified and the 30 mm-stimulus reduced by a factor of about 1.5 so that visual standard stimuli in the large discrepancy condition should have had seen lengths of about 30 and 20 mm. In visuo-haptic conditions the discrepant visual and haptic information was presented simultaneously. For the small intersensory discrepancy condition the physical stimuli were 20 and 25 mm long and optically magnified or reduced by a factor of 1.25 (seen length about 25 and 20 mm).

Each standard was paired with comparison stimuli that were distributed around the comparison's value that we expected to be perceived as equal to the standard in length (i.e., the PSE). In haptic and visual alone conditions, the expected PSEs were the standard's felt and seen lengths. In visuo-haptic conditions we only expected that the PSEs would be in-between seen and felt length, and hence used a slightly higher number of comparison stimuli around the mean of felt and seen length. Details can be found in Table 1. We accepted the unequal number of comparison stimuli, because we aimed to keep the experiment as short as possible in order to keep the children's attention engaged during the entire experiment.

**Table 1:** **Length of comparison stimuli for each standard**.

**Intersensory discrepancy and modality conditions**	**Standard stimulus (mm)**	**Comparison stimuli (mm)**	**Number of comparison stimuli (mm)**
**LARGE DISCREPANCY**			
Haptics or vision alone	20	14–26	7, steps of 2
	30	24–36	7, steps of 2
Haptics and vision	20 and 30	16–32	9, steps of 2
	30 and 20	18–34	9, steps of 2
**SMALL DISCREPANCY**			
Haptics or vision alone	20	16.25–23.75	7, steps of 1.25
	25	21.25–28.75	7, steps of 1.25
Haptics and vision	20 and 25	17.5–27.5	9, steps of 1.25
	25 and 20	17.5–27.5	9, steps of 1.25

Each standard-comparison pair was presented three times. The experiment was divided into three parts. Each part involved three blocks of trials, one block for each Modality condition (Haptics alone/Vision alone/Haptics and Vision). The order of Modality blocks was balanced across participants. Each block contained each standard-comparison pair once. The order of presentations in each block was randomized, preventing adaptation in visuo-haptic conditions. The experiment was conducted in 2–3 sessions of less than 30 min duration each.

In each trial participants first explored the standard stimulus. We chose to present standard and comparison stimuli in a fixed order, because this facilitated the participant's task, as they were required to treat standard and comparison stimuli slightly differently. Note that potential perceptual bias due to the fixed order is implicitly considered and invalidated when we calculate weights [Equation (3) and footnote 1]. In haptic alone conditions participants felt the standard for about 1–2 s. They were instructed to grasp with the thumb and the index finger of their dominant hand. Participants always touched the stimulus through a soft cloth. In visual conditions participants looked at the standard for about 2 s. In visuo-haptic conditions participants grasped the standards while looking at it, keeping visual and haptic presentation times approximately equal to the unisensory conditions, i.e., 1–2 s. In visuo-haptic conditions participants saw their finger movements through the soft cloth, so that they knew that the visual and haptic input stemmed from the same object. After having explored the standard stimulus, participants felt the comparison stimulus through a soft cloth in the other presentation box for about 1–2 s. Then, they indicated which of the two stimuli they had perceived as being larger by pointing to the corresponding presentation box. The experimenter entered the participant's response in a computer program. The experimenter also guided the participant through the experimental trials: She instructed the participants online when to explore each stimulus and when to respond while paying attention that stimulus exploration times, and the time between the exploration of the two stimuli did not exceed 2 s and response times did not exceed 10 s. Between trials the experimenter changed the stimuli and lenses in the presentation boxes as indicated by the computer program.

### Data analysis

Applying the method of constant stimuli, we acquired 21–27 responses per participant and condition. We plotted the proportion of trials in which the standard was perceived as being longer than the comparison against the length of the comparison. The PSE is defined as the amplitude of the comparison stimulus at which either stimulus is equally likely to be chosen. The JND is defined as the difference between the PSE and the amplitude of the comparison when it is judged longer than the standard 84% of the time. We fitted cumulative Gaussians to the psychometric functions using the psignifit toolbox for Matlab which implements maximum-likelihood estimation methods (Wichmann and Hill, 2001). The parameter μ of the Gaussian estimates the PSE, and, σ estimates the 84%-discrimination threshold (JND).

From the PSEs we estimated the individual weights of visual information *w*_*vemp*_ in visuo-haptic judgments for each standard stimulus; the haptic PSE_*H*_ and the visual PSE_*V*_ were estimated from group means; they were combined with the individual visuo-haptic PSE_*VH*_ as follows^1^:

(3) $$w_{vemp}=\frac{{\text{PSE}}_{VH}-{\text{PSE}}_{H}}{{\text{PSE}}_{V}-{\text{PSE}}_{H}}$$

Further, we aimed to predict the variance in the visuo-haptic conditions from the variances of haptic and visual estimates. This required, first, to estimate haptic and visual estimate variance from the JNDs, which we did as follows (left side)^2^:

(4) $$\begin{array}{l} {\text{ JND}}_{H}=\sqrt{\sigma_{h}^{2}+\sigma_{h}^{2}}\to \sigma_{h}^{2}=\frac{1}{2}{\text{JND}}_{H}{}^{2}\\ {\text{ JND}}_{V}=\sqrt{\sigma_{h}^{2}+\sigma_{v}^{2}}\to \sigma_{v}^{2}={\text{JND}}_{V}{}^{2}-\frac{1}{2}{\text{JND}}_{H}{}^{2}\\ {\text{JND}}_{V_{H}}=\sqrt{\sigma_{h}^{2}+\sigma_{vh}^{2}}\to \sigma_{vh}^{2}={\text{JND}}_{VH}{}^{2}-\frac{1}{2}{\text{JND}}_{H}{}^{2} \end{array}$$

The uppercase letters *H*, *V*, and *VH* indicate the three modality conditions, the lowercase letters refer to the modality-specific estimates derived from haptic (*h*), visual (*v*), and visuo-haptic stimuli (*vh*). Further, it is assumed that modality-specific variances are similar for different stimulus values.

Visual and haptic variance estimates were used to predict visuo-haptic variances and visual weights according to the MLE model of optimal integration [from Equation (2); (Ernst and Banks, 2002)]:

(5) $$\sigma_{vh_{\text{MLE}}}^{2}=\frac{\sigma_{v}^{2}*\sigma_{h}^{2}}{\sigma_{v}^{2}\text{+ }\sigma_{h}^{2}},w_{v_{\text{MLE}}}=\frac{\sigma_{h}^{2}}{\sigma_{h}^{2}+\sigma_{h}^{2}}$$

Further, we tested for suboptimal integration. For the weights of suboptimal integration we assumed the empirical weights *w*_*v_emp*_. Assuming that the estimates' noises are uncorrelated, visuo-haptic variance was predicted as follows (Kuschel et al., 2010):

(6) $$\sigma_{vh_{sub}}^{2}=w_{v_{emp}}^{2}\sigma_{v}^{2}+(1-w_{v_{emp}}^{2})\sigma_{h}^{2}$$

Finally, we predicted visuo-haptic variances assuming probabilistic cue switching. In this case the empirical weights *w*_*vemp*_ estimate the probability that only the visual input is used to estimate the length of a visuo-haptic stimulus. Visuo-haptic variances were predicted as follows [adapted from Kuschel et al. (2010); cf. (Nardini et al., 2008)].

(7) $$\begin{array}{l} \sigma_{vh\_switch}^{2}=w_{v_{emp}}\sigma_{v}^{2}+(1-w_{v_{emp}})\sigma_{h}^{2}+w_{v_{emp}}(1-w_{vemp})\\ \;\times \;{\left({\text{PSE}}_{V}-{\text{PSE}}_{H}\right)}^{2} \end{array}$$

Visuo-haptic variances from all three models were transformed back into JND predictions and compared to the actual JNDs. Predictions were based on individually averaged values (averaged over Stimulus Sets) and weight estimates were confined to be between 0 and 1.

## Results

### PSEs

**Figure 1:**
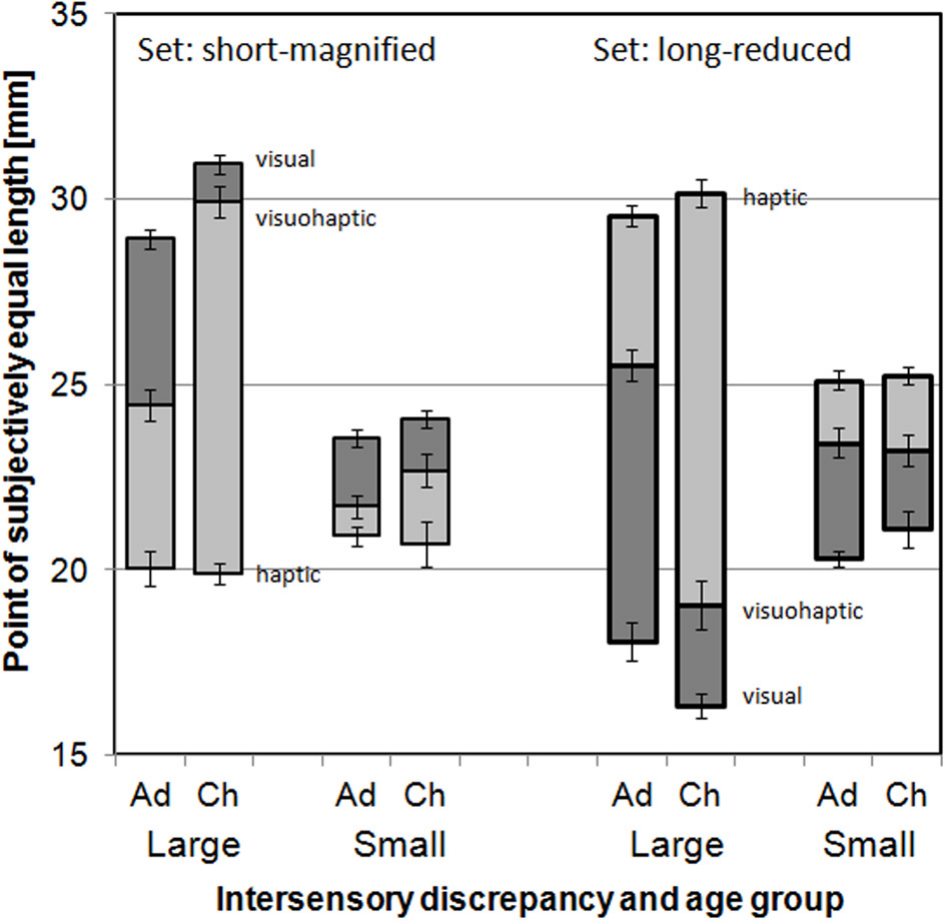
**PSEs as a function of Intersensory discrepancy, Age group (Ad[ults] vs. Ch[ildren]), and Stimulus Set**. The light gray end of the bars (lower end on the **left**, upper end on the **right**) refers to the haptics alone standard, the dark gray end to the vision alone standard and the border between light and dark gray to the visuo-haptic standard. Error bars indicate standard errors.

As should be the case, PSEs from the haptic conditions were, on average, close to the actual values of the physical stimuli, i.e., close to 20 mm for the Set “short-magnified,” and close to 25 and 30 mm, respectively, for the Set “long-reduced” (Figure 1). They did not significantly differ between age groups (*p*s > 0.10 for main effect and interactions with Age group: ANOVA of haptic PSEs with variables Intersensory discrepancy, Age group, Stimulus set). Visual PSEs indicated that the optical magnification and reduction of the physical stimuli was successful, but the PSEs did not perfectly correspond to the expected values: The physical stimulus of 20 mm length was expected to be magnified to a seen length of 25 mm in the small discrepancy condition and to 30 mm in the large discrepancy condition, but visual PSEs were, on average, 23.8 and 29.9 mm, respectively. The physical stimulus of 25 mm in the small discrepancy condition and that of 30 mm length in the large discrepancy condition were both expected to be reduced to a seen length of 20 mm, but visual PSEs were, on average, 20.7 and 17.2 mm, respectively. Additionally, the magnifying or reducing effect of the lens as assessed by the visual PSEs was slightly more pronounced for children than for adults, except for the reducing lens in the small discrepancy condition (Figure 1). An ANOVA (variables Intersensory discrepancy, Age group, Stimulus set) showed significant interactions Age group × Stimulus set, *F*_(1, 66)_ = 5.132, *p* = 0.027, and Age group × Stimulus set × Intersensory discrepancy, *F*_(1, 66)_ = 6.935, *p* = 0.011.

The deviations of the visual PSEs from the target values do, however, not limit the conclusions that can be drawn from the experiment, because further analyses were based on the visual and haptic PSEs of the stimuli, not on their physical parameters.

Finally, as can be well seen from Figure 1, PSEs from the visuo-haptic conditions were in-between the PSEs from corresponding visual and haptic conditions. This indicates some combination of the discrepant visual and haptic information. The relative shift of the visuo-haptic PSEs from the haptic toward the visual PSEs will be analyzed in the next subsection on visual weights.

### Weights

The empirical visual weights *w*_*vemp*_ were submitted to an ANOVA with the within-participant variable Stimulus set and the between-participant variables Age group and Intersensory discrepancy. The visual weights were larger for children as compared to adults, *F*_(1, 66)_ = 45.42, *p* < 0.001. Further, visual weights were, on average, larger for the large intersensory discrepancy as compared to the small one, *F*_(1, 66)_ = 18.15, *p* < 0.001, but this effect was modified by the age group [interaction Age Group × Discrepancy, *F*_(1, 66)_ = 5.20, *p* = 0.027]. Separate tests confirmed the effect of Intersensory discrepancy on the visual weight only for the children, *F*_(1, 31)_ = 21.80, *p* < 0.001, but not for the adults, *F*_(1, 35)_ = 2.00, *p* = 0.17. There were no other significant effects on the visual weights (*p*s > 0.15).

### JNDs

JND values were submitted to an ANOVA with the between-participant variables Age group and Intersensory discrepancy and the within-participant variables Modality and Stimulus set (Figure 3). On average, the JND values were larger for children than for adults, *F*_(1, 66)_ = 18.87, *p* < 0.001. Further, JNDs differed between Modalities, *F*_(2, 132)_ = 4.22, *p* = 0.017, and this effect was modified by the extent of the intersensory discrepancy, interaction: *F*_(2, 132)_ = 3.48, *p* = 0.034. With large intersensory discrepancies, visual and haptic JNDs were similar but visuo-haptic JNDs were significantly larger than both unisensory JNDs, suggesting a bisensory disadvantage. In contrast, with small intersensory discrepancies, visual JNDs were significantly larger than haptic ones, while visuo-haptic JNDs did not reliably differ from the unisensory JNDs (*post-hoc t*-tests, Bonferroni-adjusted per intersensory discrepancy, α = 5%). Numerically with small intersensory discrepancies the bisensory JNDs were in-between the unisensory ones (Figure 3). Other effects in the ANOVA were not significant (*p*s > 0.15). Note that the lack of interaction with Age group suggests that the pattern of results was similar for children and adults.

### Model predictions

Average and individual model predictions for visuo-haptic JNDs are depicted in Figures 4, 5. Although individual JND values are somewhat spread, which is a consequence of the relatively low number of data points that we were able to collect per child, the figures already provide a clear overall picture of the results. It can be seen that with the small intersensory discrepancy both the adults' and the children's data are well predicted from the model of suboptimal integration (similar average JNDs for observed and predicted values in Figure 4 corresponding to a slope close to 1 in Figure 5). In contrast, with the large intersensory discrepancy none of the models provides a good fit. The following section will report the inference statistics in detail.

#### Optimal integration

Using the model of optimal integration, we predicted optimal visual weights and optimal JNDs in bisensory conditions based on the corresponding unisensory JNDs.

**Figure 2:**
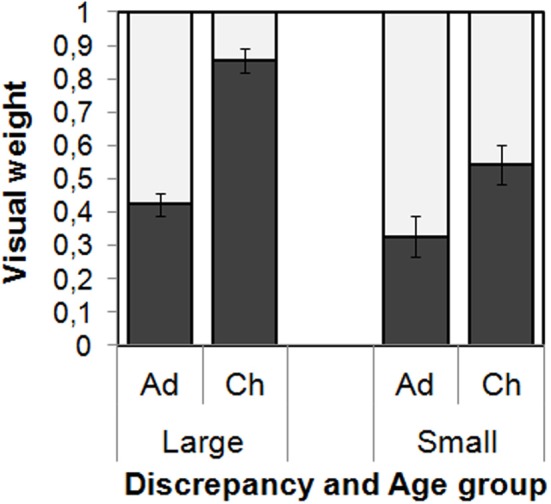
**Visual weights and standard errors as a function of Intersensory Discrepancy and Age Group (Ad[ults] vs. Ch[ildren]) averaged over the two conditions of Stimulus Set**. The dark bars indicate the visual weights. Note that weights sum up to 1, so that the length of the light bars represents the haptic weights.

In a certain number of cases (27%) optimal weight estimates were clear outlier values (>1 up to 31)^3^, which is a consequence of magnification of measurement errors through the estimation procedure. Hence, we compared predicted and observed weights using non-parametrical Wilcoxon-tests, because these are based on ranks rather than on absolute values, and we report median weights instead of means. With the large intersensory discrepancy the children's empirical weights were significantly higher than predicted from optimal integration (*Med*: 0.86 vs. 0.35, *Z* = 3.339, *p* = 0.001), while the adults' empirical weights were significantly lower than predicted (*Med*: 0.44 vs 0.68, *Z* = 2.686, *p* = 0.007; note that the median weights used in these analyses slightly differ from the averages depicted in Figure 2). With small intersensory discrepancies the same numerical trends were visible, but not significant (children: *Med*: 0.48 vs. 0.26, *Z* = 1.067, *p* = 0.268; adults: *Med*: 0.28 vs. 0.42, *Z* = 1.013, *p* = 0.311).

**Figure 3:**
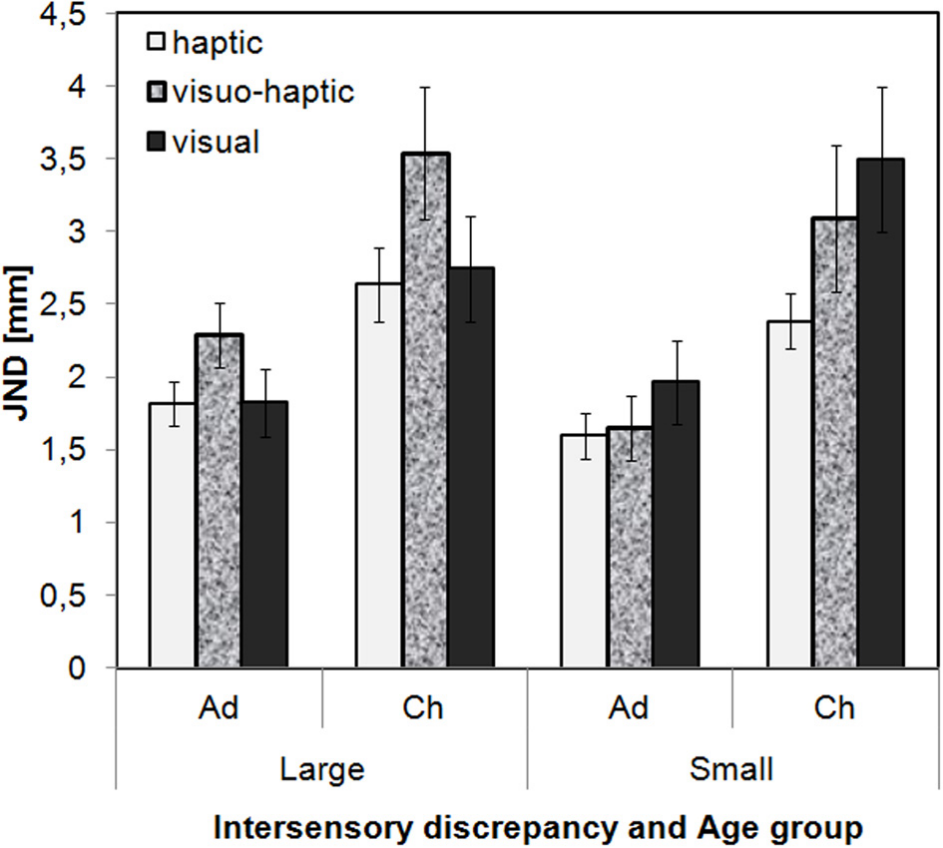
**Measured JND values and standard errors as a function of Modality, Intersensory discrepancy, and Age group (Ad[ults] vs. Ch[ildren]) averaged over the two conditions of Stimulus Set**.

**Figure 4:**
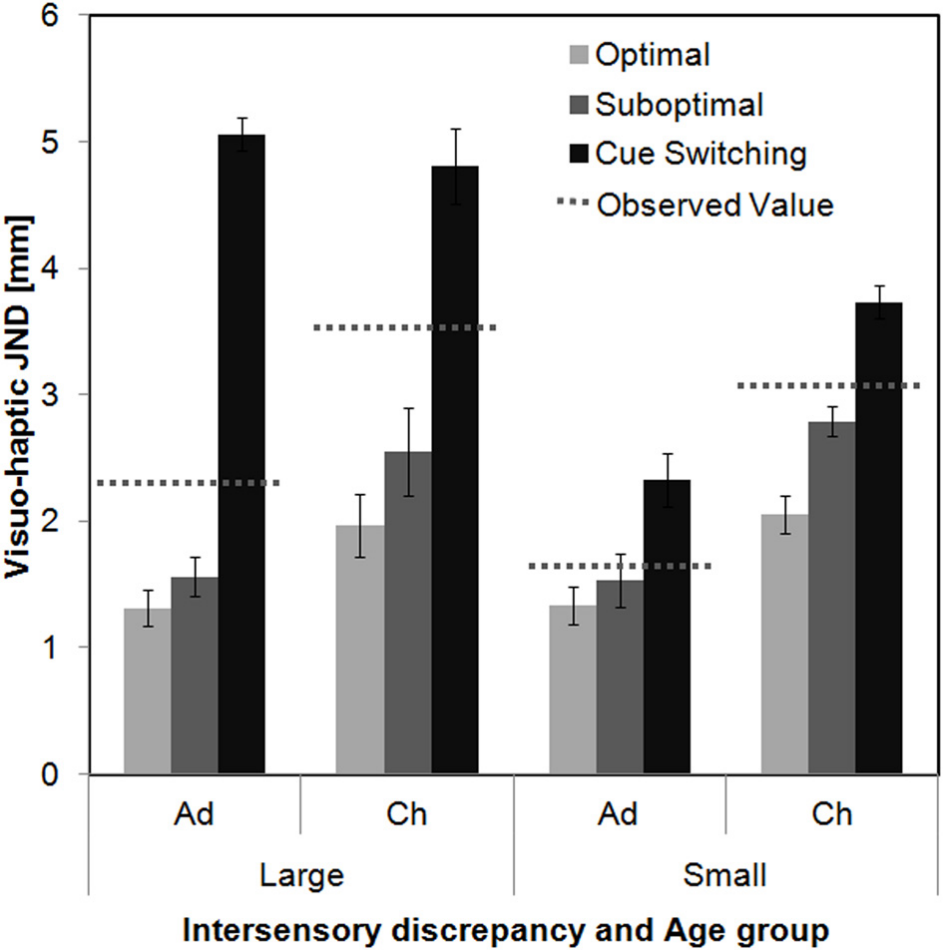
**Average predicted visuo-haptic JND values and standard errors as a function of Intersensory discrepancy and Age group (Ad[ults] vs. Ch[ildren]) collapsed over Stimulus sets**. The dotted lines represent the observed visuo-haptic JNDs.

**Figure 5:**
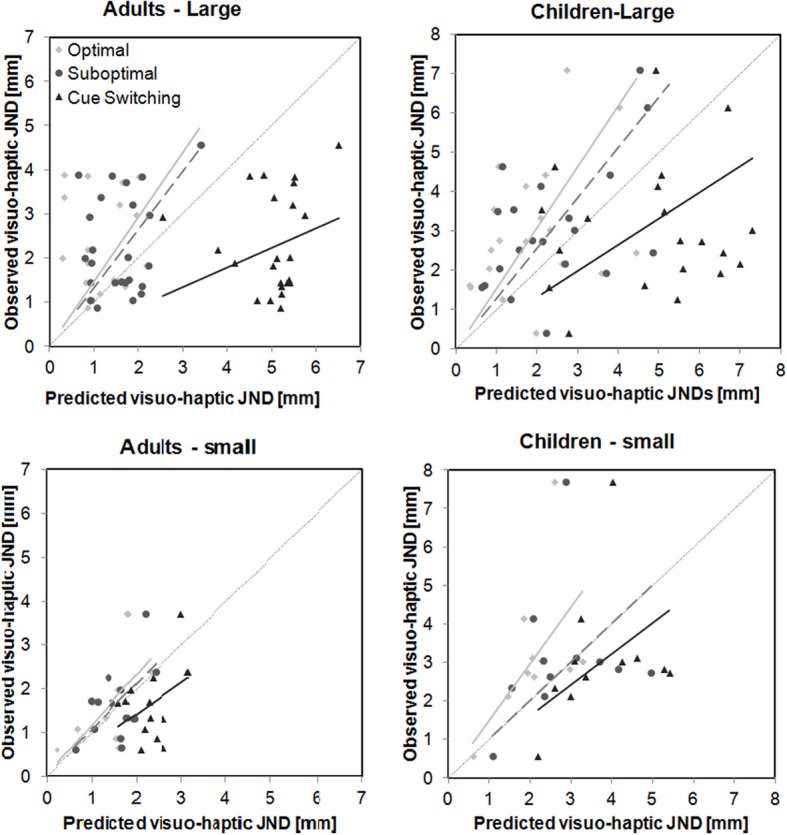
**Predicted individual visuo-haptic JND values against observed values as a function of Intersensory discrepancy and Age group**. Thick lines represent linear regressions with the constant being set to 0. Light gray diamonds and lines refer to optimal integration, middle gray circles and lines (dotted) to suboptimal integration, black triangles and lines to cue switching.

Further, we compared predicted and observed visuo-haptic JND values using two ANOVAs with the variables Age group and Value (predicted vs. observed), one analysis for each of the two intersensory discrepancies. For both discrepancies the observed JNDs were significantly larger than predicted from optimal integration, independent from Age group [Value effect, large discrepancy: *F*_(1, 44)_ = 29.87, *p* < 0.001, small discrepancy: *F*_(1, 22)_ = 8.07, *p* = 0.009; interaction Value × Age group, large: *F*_(1, 44)_ = 1.56, *p* = 0.23, small: *F*_(1, 22)_ = 2.29, *p* = 0.14]. We further tested the slopes of the linear regression (no constant, Figure 5) of observed upon predicted JNDs against a slope of 1 (=perfect prediction). These analyses confirm that optimal predictions underestimate the actual visuo-haptic JNDs for almost each discrepancy and age group [adults-large, slope 1.46, *t*_(23)_ = 2.45, *p* = 0.01; children-large, slope 1.54, *t*_(21)_ = 2.68, *p* = 0.007; children-small, slope 1. 47, *t*_(10)_ = 2.25, *p* = 0.02; exception: adults-small, slope 1.17, *t*_(12)_ = 1.09, *p* = 0.15, one-tailed].

#### Suboptimal integration

We further predicted visuo-haptic JND values under the assumption that participants integrate visual and haptic information suboptimally with the measured empirical weights. Again, two ANOVAs with the variables Age group and Value were conducted. For large intersensory discrepancies observed JNDs were significantly larger than predicted from suboptimal integration, *F*_(1, 44)_ = 16.14, *p* < 0.001, again independent from Age group [interaction Value × Age group, *F*_(1, 44)_ = 0.357, *p* = 0.55]. In contrast, for small intersensory discrepancies the observed JND values did not significantly differ from the predicted values [Value effect, *F*_(1, 22)_ =0.45, *p* = 0.009; interaction Value × Age Group, *F*_(1, 22)_ = 0.10, *p* = 0.75], indicating that these data are consistent with the assumption of a suboptimal integration both in children and adults. Slope analyses confirm that suboptimal predictions underestimate the actual visuo-haptic JNDs for large discrepancies [adults, slope 1.32, *t*_(23)_ = 2.13, *p* = 0.04; children, slope 1.28, *t*_(21)_ = 2.12, *p* = 0.046], but fit well with the data for small intersensory discrepancies [adults, slope 1.06, *t*_(12)_ = 0.48, *p* = 0.64; children, slope 1. 01, *t*_(10)_ = 0.04, *p* = 0.97].

#### Cue switching

Finally, we tested the measured visuo-haptic JNDs against predictions from cue switching, i.e., assuming that participants used either only visual or only haptic cues with probabilities that are estimated by the empirical weights. Again, two ANOVAs were conducted. For both large and small intersensory discrepancies observed JNDs were significantly smaller than predicted from cue switching, [large: *F*_(1, 44)_ = 42.93, *p* < 0.001; small: *F*_(1, 22)_ = 5.85, *p* = 0.024]. For small discrepancies the Value effect was independent from Age Group [interaction, *F*_(1, 22)_ = 0.004, *p* = 0.95], while for large discrepancies it was more pronounced for adults than for children, *F*_(1, 44)_ = 5.89, *p* < 0.019. However, the slope analyses confirm that cue switching overestimates the actual visuo-haptic JNDs for each intersensory discrepancy and age group [adults-large, slope 0.45, *t*_(23)_ = 12.24, *p* < 0.001; children-large, slope 0.66, *t*_(21)_ = 3.26, *p* = 0.002; adults-small, slope 0.71, *t*_(12)_ = 3.16, *p* = 0.004, children-small, slope 0.81, *t*_(10)_ = 1.46, *p* = 0.04; one-tailed].

## Discussion

In the present study, we investigated how adults and 6-year-old children combine seen and felt object length. We studied visuo-haptic judgments introducing a large or a small intersensory discrepancy between seen and felt length. We assessed the contribution of each sense to the bisensory judgments via the points of subjectively equal length of discrepant visuo-haptic stimuli to stimuli that were only felt. Further, we tested different models on how visual and haptic inputs are combined by comparing their predictions with the actual data.

In adults, the contribution of vision to the judgments was moderate and did not reliably depend on the magnitude of the intersensory discrepancy (average 33 and 42% for small and large discrepancies, respectively). In contrast, the children's judgments were dominated by seen length (85%) for large discrepancies, but less so for small discrepancies (54%). We conclude that children—but not adults—concentrate on a single sense, here vision, when inputs from two senses are in large conflict. However, when the inputs from the different senses seem to correspond to each other, children also can use both inputs.

But how exactly did children and adults combine the inputs from the different senses? We tested model predictions of optimal integration (using optimal weights), suboptimal integration (assuming that participants used the measured weights) and probabilistic cue switching. Integration models predict that on each presentation of a visuo-haptic stimulus participants combine both the visual and the haptic input into a single length estimate for that stimulus using a weighted average of estimates from the two senses. In contrast, probabilistic cue switching means that participants never integrate but, with a certain probability, use either only the visual input or only the haptic input to estimate the stimulus' length. Overall, with the small intersensory discrepancy, visuo-haptic JNDs tended to be in-between visual and haptic ones, but were, in contrast to the predictions of the optimal integration account (Kuschel et al., 2010), not lower than each of the unisensory JNDs. In addition, JND predictions from the model of suboptimal integration provided a good match for the data both for adults and children, whereas predictions from cue switching, and also partly predictions from optimal integration were rejected. We, hence, conclude that with the small intersensory discrepancy both adults and children integrated visual and haptic information suboptimally. In contrast, with large discrepancies, visuo-haptic JNDs were higher than predicted from optimal and suboptimal integration both in children and adults. Thereby, the bisensory JNDs were higher than the unisensory JNDs in either sense. Kuschel et al. (2010) have shown that if inputs from two senses are integrated, the variance of the bisensory estimates cannot be higher than the maximum of the variances of the two unisensory estimates. Because the present bi- and unisensory JNDs monotonically relate to the corresponding variances [cf. Equation (4)], we can, hence, conclude that with the large discrepancy neither adults nor children integrated the inputs from the two senses. However, their performance was still better than predicted from probabilistic cue switching, which we will discuss below. Taken together, we conclude that both children and adults integrated the visual and haptic input when the discrepancy between the inputs was small, but failed to integrate with large discrepancies.

Overall, the results are able to explain discrepancies between earlier findings on the development of multisensory integration. While studies on infants tend to suggest that the perception of intersensory relations and multisensory integration has an early onset, previous psychophysical studies on pre-school and school children that used psychophysical tasks similar to those used in adults found little evidence for integration in visuo-haptic tasks before the age of 8–10 years (Misceo et al., 1999; Gori et al., 2008). In these studies children rather focused on a single sense and did not combine the information from both senses. However, in these studies cues might have suggested that visual and haptic inputs did not have a common origin: either the discrepancy between the visual and the haptic input was quite large (Misceo et al., 1999) or the two inputs originated from clearly different locations (Gori et al., 2008). In the present study we explicitly tested whether a large discrepancy between the two inputs has an impact on integration. In fact, we confirmed that children do not integrate visual and haptic inputs when the discrepancies between the two inputs are large, but rather focus on a single sense (here: vision). However, we also provide evidence that with small intersensory discrepancies and cues promoting common origin, 6-year-old children integrate the two inputs, similar as adults do. Hence, we conclude that children's ability to integrate information from different senses develops earlier than suggested before. Other studies are in line with this conclusion. As an example, King et al. (2010) found multisensory-motor integration (vision and proprioception) in children aged between 7 and 13 years that was dependent on the acuity, i.e., the reliability of the single senses. The authors assume that the processes involved in multisensory integration are similar in children and adults. Furthermore, the already mentioned McGurk effect found in 4- to 10-year-old children, provides a strong case for the general abilities of pre-school children to integrate information across different senses. Massaro et al. (1986) demonstrated that children displayed the McGurk effect, but weighted the auditory information more strongly than adults. Obviously, the weighting depended on children's inferior abilities in lip-reading: children (as well as adults) who were better in lip-reading weighted the visual information more strongly than children who were less proficient in lip-reading. However, the basic integration processes were the same, according to the authors.

The finding of suboptimal integration in the small discrepancy condition seems at first glance at odds with previous findings on adult's multisensory integration as being optimal (Ernst and Banks, 2002; Alais and Burr, 2004; Helbig and Ernst, 2007a). However, as compared to many previous studies we presented comparison stimuli only to a single sense and, thus, focused on the automatic aspects of integration in the bisensory conditions while diminishing deliberate ones (cf. Ernst, 2006). The present data do not allow to decide whether integration including also deliberate aspects would have been optimal. Still the findings provide clear evidence for integration in the small discrepancy condition. It is less clear how visual and haptic inputs were used in the large discrepancy condition. Note that our results are only partly consistent with the previous literature. Like previous studies using the lens paradigm with large intersensory discrepancies (Misceo et al., 1999), we observed dominance of a single sense in children's bisensory judgments, and unambiguous contributions of both senses in adults. However, as opposed to the study by Gori et al. (2008) using bi-partite objects that found optimal integration in the adult but not in the children sample, we found no evidence of optimal integration neither in the child, nor in the adult sample. In the present study, the condition with large intersensory discrepancies led to a performance in both age-groups that rejected models of integration. The difference between the studies might originate in a particularity of the bi-partite task. Earlier work on the bi-partite task has shown that for adults task instructions regarding a shared origin of visual and haptic inputs is required to promote integration (Miller, 1972). We, hence, speculate that the explicit cognitive cues on the same origin given in the study of Gori et al. (2008) might have been differentially efficient in children and adults, while in the present study only implicit cues on the origin of the inputs were given that were similar efficient in the two groups. Hence, we observed the same behavior in adults and children in the large discrepancy condition. Thereby, it is not entirely clear how participants combined the two inputs in this context. While we can reject integration, the same is true for probabilistic cue switching as an overall explanation. However, it might be the case that the overall data reflect a mixture of different combination strategies: e.g., some individuals might have switched while others might have integrated or single individuals might have alternated between these strategies. Also fluctuations in the weights over the experiment would be able to predict high bisensory variances. The present data, however, allow no distinction between these options and further research is required on this issue. It is important to note that while large discrepancies hindered a proper integration in both adults and children, they did probably not lead to a complete single-cue strategy.

Altogether, we can conclude, however, that children combine multisensory information in similar ways as adults do, both under conditions promoting and hindering integration.

### Conflict of interest statement

The authors declare that the research was conducted in the absence of any commercial or financial relationships that could be construed as a potential conflict of interest.

## Footnotes

^1^Remember that the PSEs assessed internal length estimates $\hat{s}$_*i*_ in Equation 1, and that they do so on a single scale across modality conditions, namely as compared to haptic-only stimuli. We calculate the weights from the different PSEs only, and, thus, directly calculate the estimate's weights *w*_*i*_ in [Equation (1)]. By using this method, perceptual bias cannot not bias the calculation of estimate weights. This is an advantage over previously used methods to calculate weights, because these were partly based on physical stimulus values and require the untested assumption that perception is unbiased (Ernst and Banks, 2002; Helbig and Ernst, 2007a; Gori et al., 2008).

^2^The equations follow from the following considerations: If in a discrimination task standard and comparison stimuli are presented in the same modality, it is assumed that the squared 84%-discrimination threshold (JND) equals twice the variance of the underlying modality-specific estimates (Ernst and Banks, 2002). The underlying model is that in each discrimination trial two independent internal estimates with equal variance, one derived from the standard and one from the comparison stimulus, contribute to the overall judgment variance that is assessed by the JND. Because in the present experiment, standard and comparison are not presented in the same modality, we assume that the two corresponding estimates contribute with unequal, but modality-specific variances σ^2^ to the JNDs in the different conditions. Further, it is assumed that estimates from the two stimuli have uncorrelated noises and, thus, their variances add up.

^3^This did not occur for JND predictions, as can be seen in Figure 5. Although we conducted all analyses on predicted JNDs twice: with all data and excluding data with outlying optimal weights (>1). The conclusions were the same and we, hence, only report analyses on all data.
